# Severe Necrotizing Anterior Scleritis in Marfan Syndrome: A Case of Scleromalacia Perforans

**DOI:** 10.1002/ccr3.71138

**Published:** 2025-10-12

**Authors:** Mehrdad Motamed Shariati, Sara Ghafari

**Affiliations:** ^1^ Eye Research Center Mashhad University of Medical Sciences Mashhad Iran

**Keywords:** granulomatosis with polyangiitis, necrotizing scleritis, rheumatoid arthritis, scleromalacia perforans

## Abstract

Necrotizing scleritis is considered the most severe form of scleritis. Because of the associated risk of permanent vision loss and potential complications, aggressive treatment, which may include the use of immunosuppressives or intravenous antibiotics, based on the underlying etiology, is essential.

## Graphical abstract

Severe necrotizing scleritis.

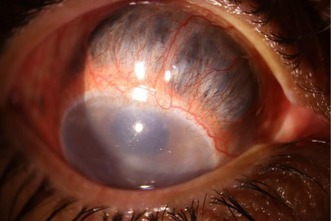

## 1 Case Presentation and Image Description

**FIGURE 1:**
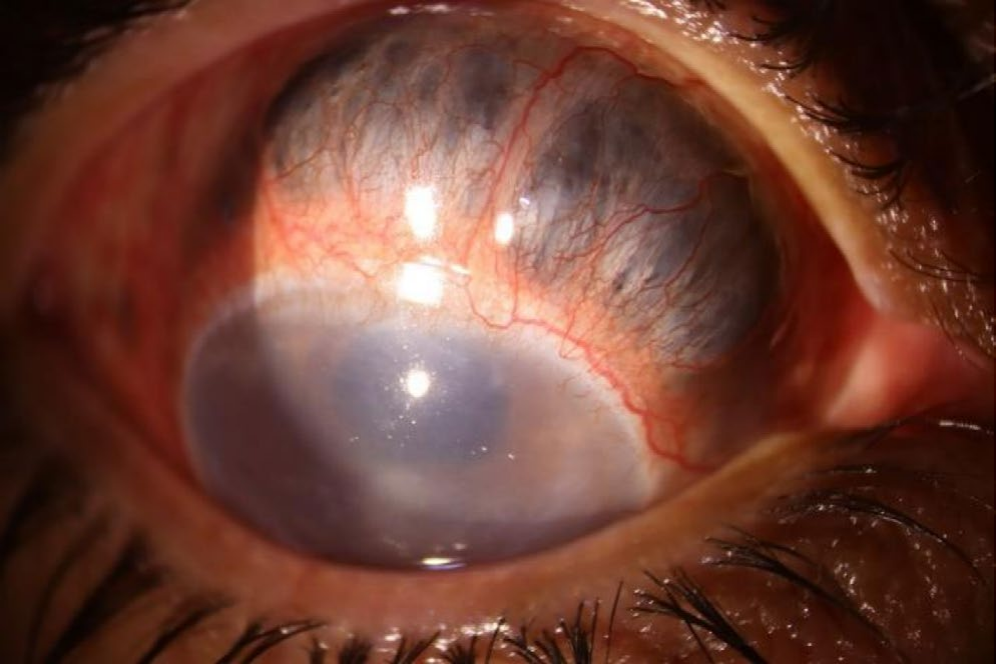
Severe scleral thinning secondary to necrotizing scleritis.

A 46‐year‐old woman with a history of Marfan syndrome presented with severe right eye pain for 4 days. Her ocular history included bilateral lensectomy for microspherophakia and a right eye vitrectomy for macula‐off retinal detachment. Examination revealed hand motion vision, positive RAPD, hypotony (5 mmHg), and a cornea with moderate stromal haziness and peripheral vascularization in the right eye. The slit lamp examination revealed scleral necrosis with adjacent inflammation (scleromalacia perforans) (Figure 1). B‐scan ultrasonography was unremarkable. Given the absence of systemic autoimmune or infectious causes on full workup, a diagnosis of idiopathic necrotizing anterior scleritis was made. The patient received intravenous methylprednisolone with planned immunosuppressive therapy.

## 2 Discussion

Necrotizing scleritis is the most destructive and vision‐threatening form of scleritis. Necrotizing scleritis can occur in systemic autoimmune disorders, systemic vasculitis, and following microbial infections. Noninfectious necrotizing scleritis has been reported in various studies to account for 1%–35% of all patients with scleritis, whereas infectious necrotizing scleritis constitutes only 5%–10% of cases of necrotizing scleritis. Rheumatoid arthritis and granulomatosis with polyangiitis remain the most identifiable systemic diseases associated with necrotizing scleritis. Infectious necrotizing scleritis can occur following planned surgical procedures, trauma to the sclera, contiguous infection from the cornea, and rarely via hematogenous spread from a distant endogenous infection [1, 2]. You can see the potential lab test investigations in Table 1.

**TABLE 1:** Systemic work‐up in necrotizing scleritis.

List of potential lab investigations in patients with scleritis	
Rheumatoid arthritis	RF ESR CRP
Granulomatosis with polyangiitis	c ANCA U/A
Microscopic polyangiitis	p ANCA U/A
Systemic lupus erythromatous	ANA ds DNA
Sarcoidosis	ACE
Tuberculosis	IGRA
Syphilis	VDRL FTA ABS
HSV/VZV	Serology
HBV	Serology
HCV	Serology

In contrast to managing other subtypes of scleritis, where a step ladder approach is recommended, non‐infectious necrotizing scleritis requires aggressive treatment from diagnosis. Systemic immunosuppressive therapy, combined with corticosteroids and either Disease‐Modifying Anti‐rheumatic Drugs (DMARDs) or biologic agents, is essential for controlling non‐infectious necrotizing scleritis [1, 2]. In our patient, the presentation of necrotizing scleritis was unilateral and developed in the context of a complex ocular history, including Marfan Syndrome, prior surgeries, vitrectomy, and scleral thinning. The absence of systemic findings or serologic markers indicative of an autoimmune disease suggests idiopathic or surgically induced necrotizing scleritis. Notably, her B‐scan ultrasonography ruled out posterior scleritis. Although no microbial etiology was identified, the diagnostic workup—including consultation with infectious disease and rheumatology specialists—was essential to initiate high‐dose corticosteroids safely.

While Marfan syndrome is not a known risk factor for necrotizing scleritis, the connective tissue fragility in Marfan patients may exacerbate the severity of scleral damage if necrotizing scleritis develops due to other causes (e.g., post‐surgical inflammation or autoimmune disease) [3]. Thus, in a Marfan patient presenting with features of scleritis, the clinician must rule out secondary inflammatory or infectious causes and manage accordingly, often with a multidisciplinary approach.

## Author Contributions

**Mehrdad Motamed Shariati:** conceptualization, methodology, supervision, writing – review and editing. **Sara Ghafari:** data curation, investigation, visualization, writing – original draft.

## Consent

Written informed consent was obtained from the patient to publish this case report and any accompanying images. A copy of the written consent is available for review by the Editor‐in‐Chief of this journal.

## Conflicts of Interest

The authors declare no conflicts of interest.

## Data Availability

The data that support the findings of this study are available from the corresponding author upon reasonable request.
